# Endoglin Haploinsufficiency Promotes Fibroblast Accumulation during Wound Healing through Akt Activation

**DOI:** 10.1371/journal.pone.0054687

**Published:** 2013-01-17

**Authors:** Miguel Pericacho, Soraya Velasco, Marta Prieto, Elena Llano, José M. López-Novoa, Alicia Rodríguez-Barbero

**Affiliations:** 1 Renal and Cardiovascular Physiopathology Unit, Instituto “Reina Sofía” de Investigación Nefrológica, Departamento de Fisiología y Farmacología, Universidad de Salamanca, Salamanca, Spain; 2 Biomedical Research Institute of Salamanca, Salamanca, Spain; University Hospital Hamburg-Eppendorf, Germany

## Abstract

Accurate regulation of dermal fibroblast function plays a crucial role in wound healing. Many fibrotic diseases are characterized by a failure to conclude normal tissue repair and the persistence of fibroblasts inside lesions. In the present study we demonstrate that endoglin haploinsufficiency promotes fibroblast accumulation during wound healing. Moreover, scars from endoglin-heterozygous (*Eng^+/−^*) mice show persisting fibroblasts 12 days after wounding, which could lead to a fibrotic scar. Endoglin haploinsufficiency results in increased proliferation and migration of primary cultured murine dermal fibroblasts (MDFs). Moreover, *Eng^+/−^* MDF have diminished responses to apoptotic signals compared with control cells. Altogether, these modifications could explain the augmented presence of fibroblasts in *Eng^+/−^* mice wounds. We demonstrate that endoglin expression regulates Akt phosphorylation and that PI3K inhibition abolishes the differences in proliferation between endoglin haploinsufficient and control cells. Finally, persistent fibroblasts in *Eng^+/−^* mice wound co-localize with a greater degree of Akt phosphorylation. Thus, endoglin haploinsufficiency seems to promote fibroblast accumulation during wound healing through the activation of the PI3K/Akt pathway. These studies open new non-Smad signaling pathway for endoglin regulating fibroblast cell function during wound healing, as new therapeutic opportunities for the treatment of fibrotic wounds.

## Introduction

Wound healing is a complex and highly coordinated process involving a number of interdependent stages including inflammation, proliferation and remodeling [1], [2]. Impairment of wound healing represents a particularly challenging clinical problem to which no efficacious treatments currently exist. Thus, understanding the complexity of the healing process is critical to resolve patient problems. In physiological remodeling, such as during dermal wound healing, fibroblast activation finishes when tissue is repaired, and activated fibroblasts disappear by apoptosis [3], [4]. However, in pathological wound healing activated fibroblasts persist and leads to fibrosis and tissue deformation, which is evident in hypertrophic scars in the fibrotic phase of scleroderma, after burn injury and in fibrosis of vital organs such as liver, heart and lung [4].

Different cells types and numerous growth factors are involved in each phase of wound healing. Among them, transforming growth factor beta (TGFβ) and its receptors, including endoglin, are essential in this process. TGFβ plays a critical role in different phases of wound healing by regulating production of extracellular matrix (ECM), proteases, chemotaxis, migration and proliferation of different cell types which regulate scar contraction, angiogenesis, granulation tissue formation, ECM remodeling and scar maduration [5].

Endoglin (CD105) is a type III co-receptor for the TGFβ receptors: TβRII, ALK1 and ALK5. Endoglin is expressed in a number of cell types including endothelial cells, monocytes, tissue macrophages, stromal cells, fibroblast, etc… and modulates TGFβ dependent responses [6], [7]. Mutations in the endoglin gene can lead to hereditary hemorrhagic telangiectasia (HHT) and defective angiogenesis [8]. Endoglin, in combination with TGFβ family members, plays an important role in regulating different cellular functions such as endothelial cell adhesion, migration and proliferation [9], [10].

Several authors have described endoglin upregulation in different fibrotic processes. Thus, endoglin expression is increased in cutaneous scleroderma fibroblasts [11], liver fibrosis [12], [13], fibroblasts isolated from strictures in Crohn's disease [14] or cardiac fibroblasts developing fibrosis [15]. Moreover, endoglin is upregulated in chronic progressive renal disease [16] and in several models of renal fibrosis [17], [18], [19]. Endoglin is mainly considered as an antifibrotic molecule. Several studies show that endoglin counteracts TGFβ1-dependent responses, such as increased expression of extracellular matrix components, including PAI-1, collagen and fibronectin [20], [21], [22], [23]. It has been described that endoglin could exert this antifibrotic role modulating TGFβ1 signaling through pro-proliferative ALK1-Smad1/5 pathway instead pro-fibrotic ALK5-Smad2/3 pathway [24], [25], [26]. These results have been confirmed in cultured fibroblasts as endoglin overexpression leads to a diminution of ECM proteins expression [14], [27]. However, some controversy exists as other authors have described profibrotic effects of endoglin expression [13], [28], [29]. These results suggest that the specific role of endoglin depends on the cell type, environmental conditions or the fibrosis model assessed. Nevertheless the importance of the study of the role of endoglin in fibrotic processes is clear.

To evaluate whether endoglin might be involved in post-wound healing fibrosis, we used endoglin-heterozygous mice (*Eng^+/−^*), since mice lacking endoglin (*Eng^−/−^*) die from cardiovascular defects at mid-gestation [30], [31], [32]. To investigate the mechanism of action of endoglin, we assessed the response of endoglin-heterozygous mice and dermal fibroblasts derived from them to wounding both *in vivo* and *in vitro*. Our results revealed that endoglin acts as an essential component for the accurate completion of tissue repair by its ability to decrease the number of fibroblasts in wounds through its capacity to block Akt activation.

## Materials and Methods

### Animals and wound model

All procedures were approved by the Committee for the Care and Use of Animals of the University of Salamanca and complied with the Guide for the Care and Use of Laboratory Animals [33]. *Eng^+/+^* and *Eng^+/−^* mice were obtained as previously described [31]. The animals were a generous gift from Michelle Letarte (Hospital for Sick Children, Toronto, Canada), and they were cared for and genotyped as previously described [34].

Eighteen *Eng^+/+^* and eighteen *Eng^+/−^* 10-week-old animals were used for the *in vivo* studies. Mice were anesthetized with isoflorane and two 5 mm of diameter excisional wound were made in the shaved middorsal skin. An aseptic technique was used for all surgical manipulations performed the mice.

### Histology and immunohistofluorescence

For histological analysis, 6-day and 12-day wounds were harvested and fixed in cold 4% buffered formalin, dehydrated, bisected, mounted in paraffin, and sectioned for histology and immunohistofluorescence. Heat-induced antigen retrieval was performed in citrate buffer (pH 9.00). The primary antibodies used were mouse monoclonal anti-α-SMA (Sigma, at 1∶300 dilution) and rabbit polyclonal anti-phospho-Akt (Ser^473^) (Cell Signaling, at 1∶10 dilution). Following washes in PBS, sections were incubated with fluorescent-conjugated secondary antibodies (anti-mouse FITC and anti-rabbit Cy3 respectively) at room temperature. Slides were mounted in Vectashield (Vector Laboratories) after nucleus staining with DAPI. All images were obtained using a confocal microscope (Leica) with identical parameters for intensity, pinhole aperture, etc.

### Cell cultures

Primary cultured murine dermal fibroblasts (MDF) were obtained from *Eng^+/+^* and *Eng^+/−^* mice. Animals were euthanized and the shaved skin was placed in a sterile flask containing 0.25% trypsin (Sigma) in PBS and incubated overnight at 4°C, after which the epidermis was separated from the dermis. The dermis was subjected to further digestion with 0.25% collagenase (Sigma) in PBS for 2–3 hours at 37°C. Cells were collected by centrifugation, washed once with PBS, and plated on 100 mm Petri dishes.

MDF and NIH3T3 (ATCC) cells were cultured in DMEM (Gibco) supplemented with 10% FBS (Gibco) and 100 U/ml of penicillin-streptomycin at 37°C in a 5% CO_2_ atmosphere. All experiments were performed in the presence of 10% FBS and MDF were used at passage four.

### Reagents and antibodies

Purified TGFβ1 was purchased from R&D Systems. The PI3K inhibitor LY294002 was from Sigma. Antibodies against Smad1, Smad2/3, Id-1 and Akt were from SantaCruz Biotechnology. Antibodies against phosphorylated Smad1/3, phosphorylated Akt (Ser^473^) and phosphorylated Akt (Thr^308^) were from Cell Signaling. Antibodies against fibronectin and collagen I were from Chemicon. The antibody against PCNA was from BD Pharmingen. The antibody against α-SMA was from Sigma. The antibody against phosphorylated Smad2 was generated as described previously [35]. The antibodies against endoglin have been described previously and were MJ7/18 for murine endoglin [36] and TEA1/58-1 for human endoglin [37].

### Retroviral transduction

293T cells (3×10^6^) were plated on a 10 cm dish, incubated overnight, and then co-transfected according to the calcium phosphate precipitation method with 10 μg of pCL-Eco plasmid containing the gag, pol and env viral proteins and 10 μg of a pBabe-puro retroviral vector containing the human endoglin gene (15 hr at 37°C). After 48 hr, the virus-containing medium was filtered (0.45 μm filter, Millipore) and supplemented with 4 μg/ml polybrene (Sigma) (first supernatant). Viral supernatants were collected for an additional 8 hr, as before (second supernatant). NIH3T3 cells were plated at 8×10^5^ cells per 100 mm dish and incubated overnight. For infection, the culture medium was replaced with the appropriate first supernatant and incubated for 8 hr at 37°C. The infection process was repeated using the second supernatant. Twenty-four hours after infection, infected cells were selected for 3 days in the presence of 2 μg/ml puromycin, and plated on the 5^th^ day post-infection for the corresponding assays.

### Immunofluorescence staining

Immunofluorescence staining was performed as previously described [23]. Cells were plated onto glass coverslips, fixed, permeabilized, and incubated with primary antibodies for 1 hour. After washing, the cells were incubated with the appropriate fluorescence-conjugated secondary antibodies (Molecular Probes) for 1 hour. Slides only incubated with the secondary antibodies were used as controls for non-specific signal. Cells were washed in 0.2% BSA-PBS, briefly rinsed in 2 μM Hoechst 33258 reagent (Invitrogen) to stain nuclei, and mounted with ProLong® antifade (Invitrogen). Stained cells were photographed with a Nikon Eclipse TE 2000-U confocal microscope.

### Western blot analyses

Western blot analyses were essentially performed as described previously [23]. Cells were lysed in ice-cold lysis buffer and protein concentrations were determined (Bradford, Bio-Rad). Protein samples were separated by SDS-PAGE, blotted onto PVDF membranes, and incubated with the primary antibody. Following incubation with horseradish peroxidase-conjugated secondary antibody, the bands were visualized with a luminol-based detection system with p-iodophenol enhancement. Anti-tubulin antibody was used to confirm equal loading of protein in each lane. Some membranes were re-probed with several antibodies using a stripping solution (Chemicon) and following the manufacturer's instructions.

### RT-PCR analysis

Total RNA was isolated using Nucleospin RNAII (Macherey-Nagel), according to the manufacturer's instructions. Single-strand cDNA was generated from 2 μg of total RNA using poly-dT as primer with M-MLV reverse transcriptase (Promega). For RT-PCR, 1 μl of cDNA was used in a standard 50-μl PCR mixture with 400 nM of each primer and 2 U of FastStart Taq DNA polymerase (Roche). The PCR products were separated by electrophoresis on a 1% agarose gel and visualized by SybrSafe (Invitrogen) staining.

Quantitative RT-PCR was performed in triplicate. Each 20 μl reaction contained 1 μl of cDNA, 400 nM of each primer, and 1x iQ SybrGreen Supermix (Bio-Rad). Standard curves were run for each transcript to ensure exponential amplification and to rule out non-specific amplification. Gene expression was normalized to RPS13 expression. The reactions were run on an iQ5 Real-time PCR detection system (Bio-Rad). The specific primers used for PCR are described in Table 1.

**Table 1:** Sequences of primers.

Gene	Sense primer (5′-3′)	Anti-sense primer (5′-3′)
Eng	GACTTCAGATTGGAATACCTTGG	CAGTGCCGTGTCTTTCTGTAAT
Collagen Iα	TGTTGCTGAAAGACTACCTCGT	CCTCCCATGTTAAATAGCACCT
Fibronectin	ATGTGGACCCCTCCTGATAGT	GCCCAGTGATTTCAGCAAAGG
PAI-1	GCTGCACTGGTGACTCACTT	AGGAGCTGGCTGTTTCTTTC
GAPDH	TGAAGGTCGGTGTGAACGGATTTGGC	CATGTAGGCCATGAGGTCCACCAC
RPS13	GATGCTAAATTCCGCCTGAT	TAGAGCAGAGGCTGTGGATG

### Plasmids, transfection, and luciferase reporter assays

The expression plasmids for human endoglin (*ENG*) have been described previously [38], [39]. ON-TARGETplus SMARTpool siRNA against *ENG* and control siRNA were obtained from Dharmacon. The TGFβ-responsive vectors used as reporters were the ALK5-Smad3-specific (CAGA)_12_-Luc [40] , the specific Smad2-responsive Fast/pAre-Luc [41], and p(Bre)_2_-Luc, which contains ALK1-specific response elements [42]. In the luciferase assays, the expression plasmid pRL-TK vector containing the Renilla luciferase gene (Promega) served as an internal control to correct transfection efficiency. Cells were transfected using Lipofectamine 2000 (Invitrogen) for 5 hours, according to the manufacturer's instructions. Luciferase and Renilla activities were measured using a dual-reporter assay kit (Promega).

### Proliferation assays

For crystal-violet assays, 5000 cells were seeded on a 24-well plate and incubated in 10% FCS medium with or without additional agonists. After the indicated times, cells were stained with crystal-violet and, upon solubilization, the amount of dye taken up by the cells was quantitated in a plate reader. Cell number was estimated based on absorbance at 595 nm. MDF proliferation was also determined by a colorimetric Cell Proliferation ELISA (Roche) following the recommendations of the manufacturer. This ELISA is based on the measurement of BrdU incorporation during DNA synthesis in replicating (cycling) cells. NIH3T3 cell proliferation was analyzed with the MTT-based assay (Sigma). The cell cycle profile was analyzed by flow cytometry.

### Migration assays

For wound healing assays, confluent monolayers were wounded using a sterile pipette tip and the extent of wound closure was determined along 24 hours by calculating the migrated distance/total wound distance. Furthermore, cell invasion was assessed through an 8.0 μm-pore membrane. MDF cells were seeded in the upper chamber of the insert in 2% FCS medium and allowed to migrate to 10% FCS medium, placed in the lower chamber, for 24 hours. Migrated cells were determined by crystal-violet assay.

### Caspase activity

To determine DEVDase activity, 20000 cells were seeded on black 96-wells plates for 24 h. Cells were treated for 3 h with vehicle (Control) or a mix of 2.5 µg/ml anti-Fas antibody (Upstate-Millipore) plus 250 µg/ml cycloheximide (Apoptosis). DEVDase activity was determined with SensoLyte™ Homogeneus Rh110 Caspase-3/7 Assay Kit (Anaspec) following the recommendations of the manufacturer.

### Statistical analyses

All numerical data are presented as means ± S.E.M and were analyzed by two-way ANOVA and Student's *t*-test. All statistical tests were performed using GraphPad Prism 5 software.

## Results

### Activated fibroblast persistence in the wounds of *Eng^+/−^* mice

**Figure 1:**
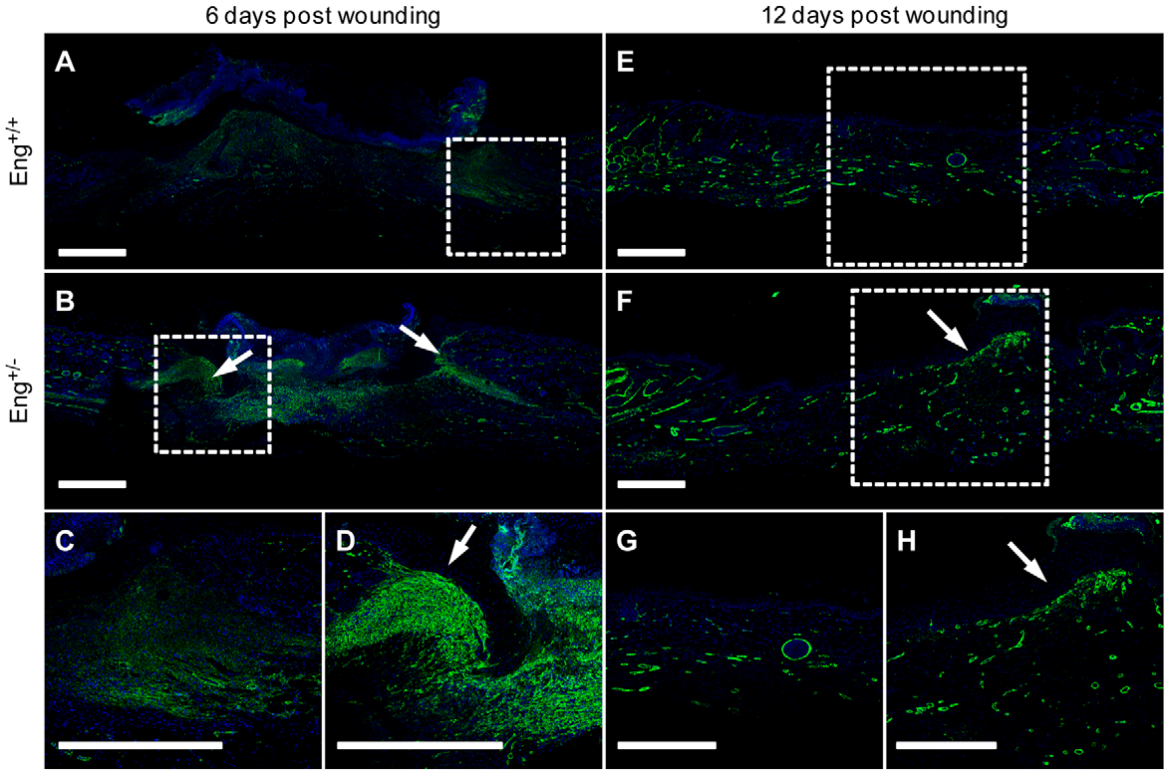
Histological analysis of activated fibroblasts presence during Eng^+/−^ wound healing. (**A** and **B**) Immunofluorescence staining of alpha-smooth muscle actin (α-SMA) on Formalin-fixed Parafin-embedded wounds from *Eng^+/+^* (**A**) and *Eng^+/−^* (**B**) mice at day 6. (**C** and **D**) Granulation tissue on wounds edges from *Eng^+/+^* (**C**) and *Eng^+/−^* (**D**) mice in more details. (**E** and **F**) Immunofluorescence staining of α-SMA on Formalin-fixed Parafin-embedded scars from *Eng^+/+^* (**E**) and *Eng^+/−^* (**F**) mice at day 12. (**G** and **H**) *Eng^+/+^* (**G**) and *Eng^+/−^* (**H**) day-12 scars magnification. Bars = 500 mm. Arrow = Activated fibroblasts accumulation. A representative image from four independent experiments is shown.

Immunohistological analyses of 6-day post-wounding skin sections revealed the presence of activated fibroblasts (α-SMA positive cells) in the granulation tissue of both genotypes. However, a higher number of activated fibroblasts were observed in granulation tissues from *Eng^+/−^* mice than in the control animals (Figure 1A–D). In 12-day post-wounding skin sections from *Eng^+/+^* mice, α-SMA expression was limited to a few resident fibroblasts, similar to normal skin (Figure 1E and 1G). However, in wounds of *Eng^+/−^* mice activated fibroblasts persisted close to the epidermis (Figure 1F and 1H). In both 6-day and 12-day skin sections we can also detect blood vessels because of the staining of the smooth muscle cells. In order to perform an in-depth analysis of these endoglin-mediated differences in activated fibroblast accumulation, we developed a primary culture of murine dermal fibroblasts (MDF) from *Eng^+/+^* and *Eng^+/−^* mice.

### Endoglin heterozygosity promotes extracellular matrix proteins synthesis

In order to evaluate the effect of endoglin deficiency in extracellular matrix (ECM) deposition, we analyzed the expression of ECM-related genes in primary cultured Murine Dermal Fibroblasts (MDF).

**Figure 2:**
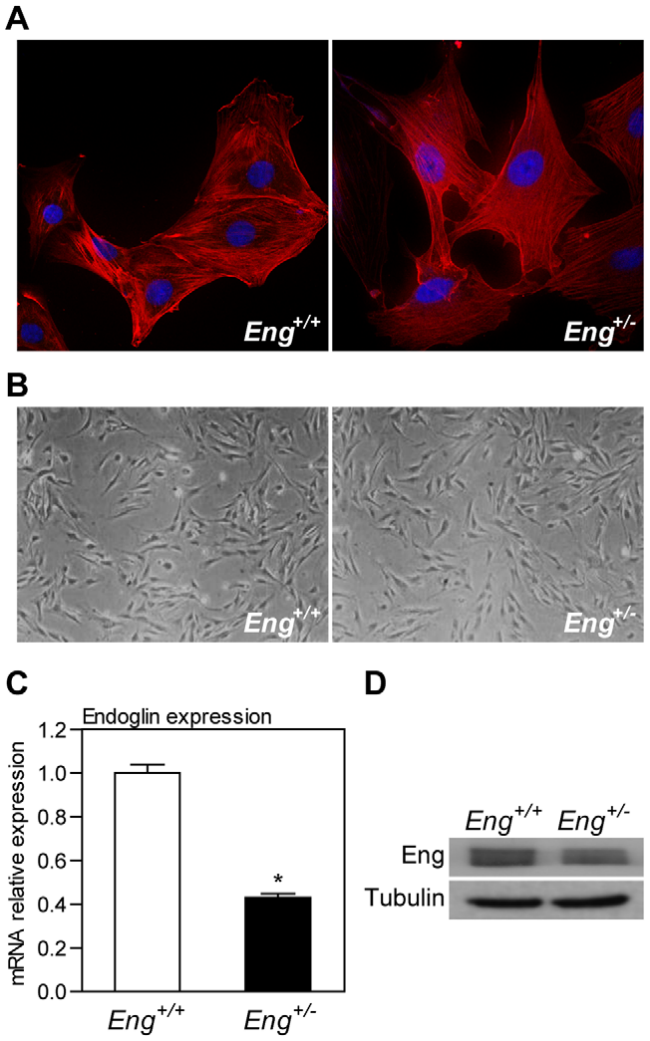
Characterization of primary cultured murine dermal fibroblasts (MDF) from endoglin heterozygous mice. (**A**) Immunofluorescence analysis of α-SMA expression in *Eng^+/+^* and *Eng^+/−^* MDF. (**B**) Phase contrast analysis of *Eng^+/+^* and *Eng^+/−^* MDF morphology. (**C** and **D**) Analysis of relative endoglin expression between *Eng^+/+^* and *Eng^+/−^* MDF by quantitative RT-PCR (**C**) and western blot (**D**). Mean+SEM is represented (n = 3). * p<0.05 -significance of the difference between cells, Student's *t*-test. A representative blot from three independent experiments is shown.

**Figure 3:**
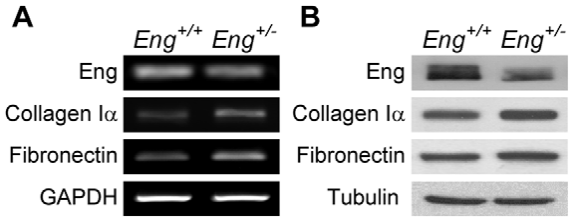
Effect of endoglin haploinsufficiency on extracellular matrix formation. (**A**) Synthesis of collagen Iα and fibronectin by *Eng^+/+^* and *Eng^+/−^* MDF were analyzed by RT-PCR. GAPDH was used as housekeeping (**B**) Synthesis of collagen Iα and fibronectin were also evaluated by western blot in total protein extracts from *Eng^+/+^* and *Eng^+/−^* MDF using Tubulin as loading control. A representative blot from three independent experiments is shown.

MDF cultures were established using dermis from either control or endoglin-heterozygous mice. After two passages in culture, we obtained a homogeneous monolayer without significant morphological differences between *Eng^+/+^* and *Eng^+/−^* MDF in phase-contrast microscopy studies (Figure 2B). We found no differences between both genotypes in expression or disposition of α-SMA, a defining characteristic of activated fibroblasts (Figure 2A). Quantitative RT-PCR and western blot analyses revealed reduced endoglin mRNA and protein levels in *Eng^+/−^* as compared to *Eng^+/+^* MDF, respectively (Figure 2C and D).RT-PCR analysis showed that *Eng^+/−^-*derived MDF presents higher expression of collagen Iα and fibronectin than *Eng^+/+^* MDF (Figure 3A). We confirmed by western blot analysis that this difference occurs also at the protein level (Figure 3B).

### Endoglin haploinsufficiency increases proliferation of MDF

Since proliferation, persistence and migration of activated fibroblasts are key events for fibroblast accumulation in granulation tissue, we analyzed whether endoglin might be modulating these properties in primary cultured dermal fibroblasts. We and others have previously reported the effect of endoglin expression on proliferation and migration in several cell types [24], [26], [43], [44].

**Figure 4:**
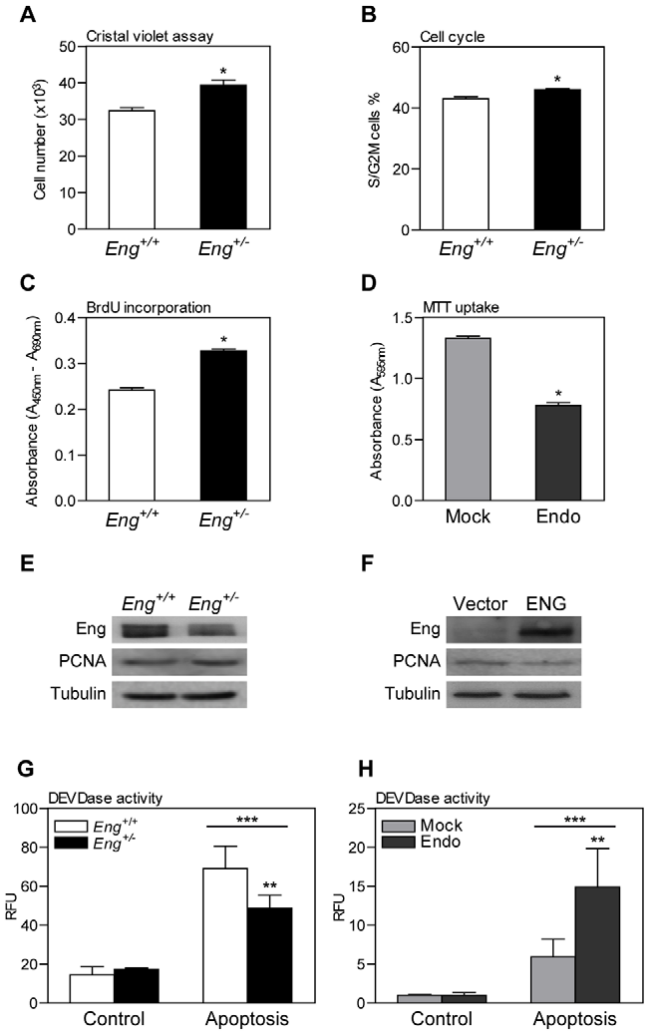
Effect of endoglin expression on fibroblast proliferation and apoptosis. (**A–C**) Endoglin haploinsufficiency effect on MDF proliferation was estimated by crystal violet assay (**A**), cell cycle analysis (**B**) and BrdU incorporation (**C**) in proliferative conditions (10% FCS). NIH3T3 fibroblasts overexpressing human endoglin (Endo) and infected with empty vector (Mock) were used to confirm the effect of endoglin expression on fibroblast proliferation. NIH3T3 proliferation was assessed by MTT (**D**). (**E and F**) PCNA expression analyzed by western blot was compared between *Eng^+/+^* and *Eng^+/−^* MDF (**E**) and on *Eng^+/−^* MDF transfected with endoglin (ENG) or the empty vector (**F**). (**G** and **H**) *Eng^+/+^* and *Eng^+/−^* MDF (**G**) and Mock and Endo NIH3T3 (**H**) *in vitro* responses to apoptotic stimulus were determined by measuring DEVDase (caspase) activity after treatment with a mix of 2.5 µg/ml anti-Fas antibody plus 250 µg/ml cycloheximide. Mean+SEM is represented (n = 3). * p<0.05-significance of the difference between cells, Student's *t*-test. ** p<0.05-significance of the difference between cells in apoptosis conditions, *** p<0.05-significance of the difference between apoptosis treatment and control conditions, Two-way ANOVA. A representative blot from three independent experiments is shown.

To investigate how affect the reduce levels of endoglin in proliferation of MDF cells, we determined cellular proliferation rates using crystal-violet staining. This analysis showed that *Eng^+/−^* cells proliferated significantly faster than wild-type MDF (Figure 4A). In addition, analysis of the cell cycle by flow cytometry revealed a greater percentage of *Eng^+/−^* cells in S+G2/M phases as compared to *Eng^+/+^* (Figure 4B). We also analyzed the cell proliferation measuring incorporation of BrdU during DNA synthesis in cycling cells. *Eng^+/−^* cells proliferate significantly more than wild-type MDF (Figure 4C). Western blot analyses showed that *Eng^+/−^* cells had a higher expression of proliferating cell nuclear antigen (PCNA) than *Eng^+/+^* cells (Figure 4E, left panel). These effects on cellular proliferation were directly dependent on a low endoglin expression, since transfection of *Eng^+/−^* MDF with endoglin (ENG) resulted in a decreased expression of PCNA (Figure 4F, right panel).

To further assess the effect of endoglin expression on fibroblasts behavior, we expressed human endoglin (hENG) in NIH3T3 fibroblasts using retroviral infection. This model allowed us to investigate whether this effect was directly dependent on endoglin expression and whether it was consistent in other fibroblast cell line. Consistent with our previous results, MTT assays revealed that endoglin overexpression inhibited NIH3T3 cell proliferation (Figure 4D).

### Endoglin haploinsufficiency MDF presents diminished apoptosis response

After wound closure, activated fibroblasts should disappear by apoptosis. However, we have previously described a persistence of activated fibroblasts in wounds of *Eng^+/−^* mice that could lead to fibrosis and tissue deformation. This persistence could be explained whether *Eng^+/−^* activated fibroblasts have diminished responses to apoptotic signals compared with control cells. To test this hypothesis we treated MDF with a combination of an activator of the extrinsic (anti-Fas antibody) and an activator of the intrinsic pathway (cycloheximide) of apoptosis an analyzed Caspase-3/7 activity. *Eng^+/−^* cells present significantly less DEVDase activity than *Eng^+/+^* cells after apoptotic treatment (Figure 4G). Endoglin overexpression in NIH3T3 fibroblasts result in a significant increase of Caspase-3/7 activity in response to apoptotic stimulus.

### Endoglin haploinsufficiency increases migration of MDF

**Figure 5:**
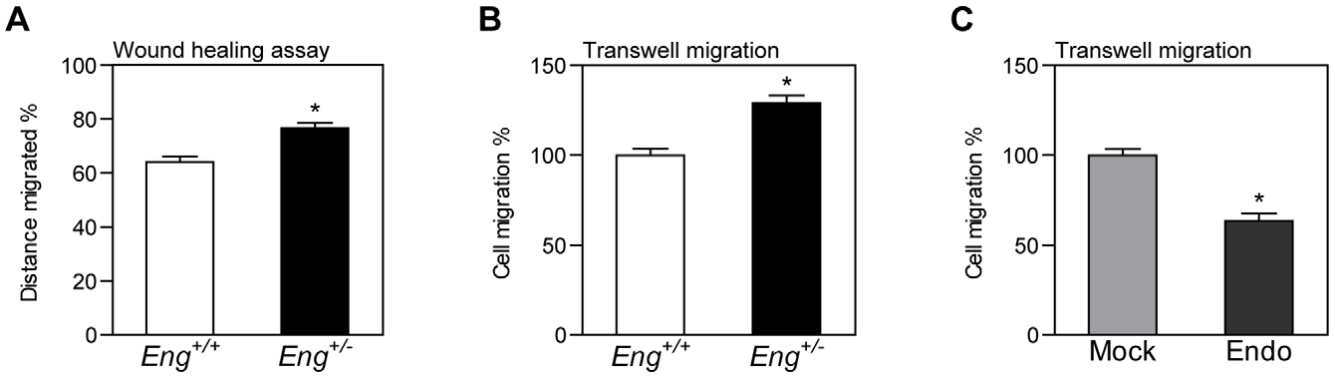
Effect of endoglin expression on fibroblast migration. (**A** and **B**) *Eng^+/+^* and *Eng^+/−^* MDF *in vitro* migration was analyzed by wound healing assay (**A**) and 8-mm pore transwell migration (**B**). (**C**) Migration of NIH3T3 fibroblasts overexpressing human endoglin (Endo) and infected with empty vector (Mock) was assessed by transwell assay. * p<0.05-significance of the difference between cells, Student's *t*-test.

Dermal fibroblasts migration was analyzed with two independent techniques, which afforded similar results. Confluent monolayers were damaged by a scratch-wound assay and the cells were allowed to migrate for 24 hours. *Eng^+/−^* cells migrated significantly farther than *Eng^+/+^* cells (Figure 5A). Time-lapse videos revealed that monolayer repair occurred as a result of fibroblast motility instead of proliferation (data not shown). Moreover, similar results were obtained in transwell migration assays (Figure 5B). Endoglin overexpression also inhibited NIH3T3 cell migration analyzed with transwell migration assays (Figure 5C).

### TGFβ1 signaling through the Smad and MAPK pathways in fibroblasts are unaffected by endoglin haploinsufficiency

**Figure 6:**
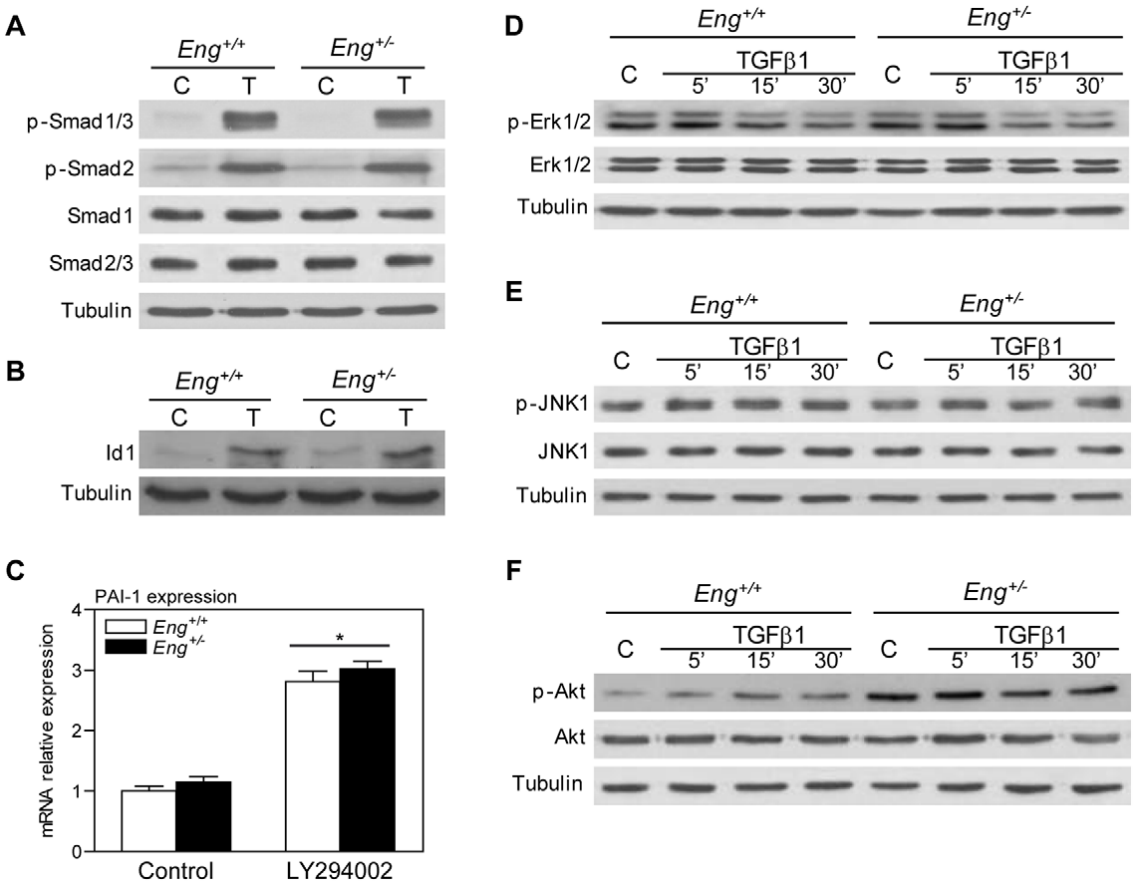
Effect of endoglin haploinsufficiency on TGFβ1 signaling pathways. (**A**) *Eng^+/+^* and *Eng^+/−^* MDF were stimulated with 1 ng/ml TGFβ1 for 30 minutes. Total protein extracts were analyzed by western blot with anti-phospho-Smad1/3, anti-phospho-Smad2, anti-Smad1 and anti-Smad2/3 (**B**). Id1 expression was assessed by western blot after 1 hour TGFβ1 treatment. (**C**) TGFβ1-induced PAI-1 expression increment was analyzed by quantitative RT-PCR. A representative experiment of three independent experiments using triplicate samples is shown. (**D**–**F**) MAPKs and PI3K/Akt pathways were analyzed after stimulation with 1 ng/ml TGFβ1 for 5, 15 and 30 minutes. Total proteins extracts were resolved by western blot and incubated with anti-phospho-Erk1/2 and anti-Erk1/2 (**D**), anti-phospho-JNK1 and anti-JNK1 (**E**) and anti-phospho-Akt and anti-Akt (**F**) respectively. Tubulin was used as loading control. Mean+SEM is represented (n = 3). * p<0.05-significance of the difference between TGFβ1 treatment and control conditions, Two-way ANOVA. A representative blot from three independent experiments is shown.

It has been proposed that endoglin modifies cellular properties such as proliferation and migration by modulating TGFβ signaling through the ALK1-Smad1, ALK5-Smad2/3 or MAPKs pathways [24], [45], [46], [47], [48], [49], [50]. Thus, first we analyzed the effect of endoglin haploinsufficiency on ALK1-Smad1 and ALK5-Smad2/3 signaling pathway activation in response to TGFβ1 treatment in dermal fibroblasts. Stimulation of MDF with TGFβ1 for 30 minutes resulted in Smad1, Smad2 and Smad3 phosphorylation (Figure 6A) without significant differences between *Eng^+/+^* and *Eng^+/−^* MDF. Moreover, TGFβ1-induced Id1 protein and PAI-1 mRNA expression was equal in endoglin-haploinsufficient and wild-type cells (Figure 6B and C). These findings suggest that endoglin expression does not modulate TGFβ1 signaling through the Smad pathways in dermal fibroblasts.

We also analyzed the effect of endoglin expression in other signaling pathways such as MAPKs or PI3K/Akt, which have been shown to be activated by TGFβ1. Stimulation with TGFβ1 resulted in an increased phosphorylation of Erk1 and Erk2 after 5 minutes of treatment (Figure 6D). TGFβ1 treatment also resulted in JNK1 activation (Figure 6E). However, there were no differences between *Eng^+/+^* and *Eng^+/−^* MDF in TGFβ1-mediated activation of these pathways. We failed to detect p38 phosphorylation in MDF after TGFβ1 treatment (data not shown).

### Endoglin expression modifies Akt activation

**Figure 7:**
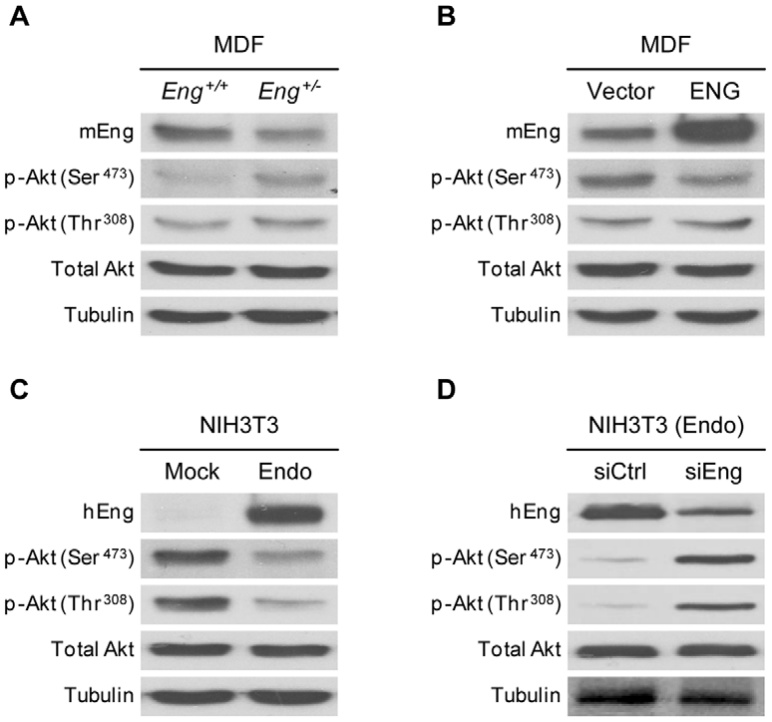
Effect of endoglin expression on Akt phosphorylation. (**A**) Ser^473^ and Thr^308^ Akt phosphorilation and Akt expression were analyzed by western blot in total protein extracts from *Eng^+/+^* and *Eng^+/−^* MDF. (**B**–**D**) Similar analysis were carried on in *Eng^+/−^* MDF transiently transfected with ENG or empty vector (**B**); NIH3T3 overexpressing by retroviral infection endoglin (Endo) and its controls (Mock) (**C**); and Endo fibroblasts transfected with anti-Eng siRNA (**D**). Tubulin was used as loading control. A representative blot from three independent experiments is shown.

Under basal conditions (10% FCS), *Eng^+/−^* cells showed a higher degree of Akt activation than control cells. TGFβ1 treatment stimulated Akt phosphorylation in both cell types, maintaining the previously observed differences between *Eng^+/+^* and *Eng^+/−^* MDF (Figure 6F). As stated, western blot analyses of MDF revealed that basal Akt activation was increased in endoglin-heterozygous cells. Akt activation is determined by multisite phosphorylation, with two main phosphorylation sites: Thr^308^ and Ser^473^ [51]. We found differences in both Akt phosphorylation sites, although greatest increase was seen in Ser^473^ phosphorylation (Figure 7A). In order to assess the direct relationship between endoglin expression and Akt activation, we overexpressed endoglin in MDFs (Figure 7B, upper panel) and analyzed Akt activation. MDF transiently transfected with endoglin showed a lower degree of Akt activation than cells transfected with empty vector (Figure 7B). Furthermore, we analyzed Akt activation in endoglin-infected NIH3T3 fibroblasts. Endoglin overexpression resulted in an important decrease in the Akt phosphorylation of both Thr^308^ and Ser^473^ (Figure 7C). Transient transfection of these cells with a siRNA for endoglin decreased endoglin expression and increased Akt phosphorylation (Figure 7D). Thus, the amount of endoglin expression seems to modulate basal Akt activation in fibroblasts.

### Akt mediates differential cell proliferation due to endoglin expression

**Figure 8:**
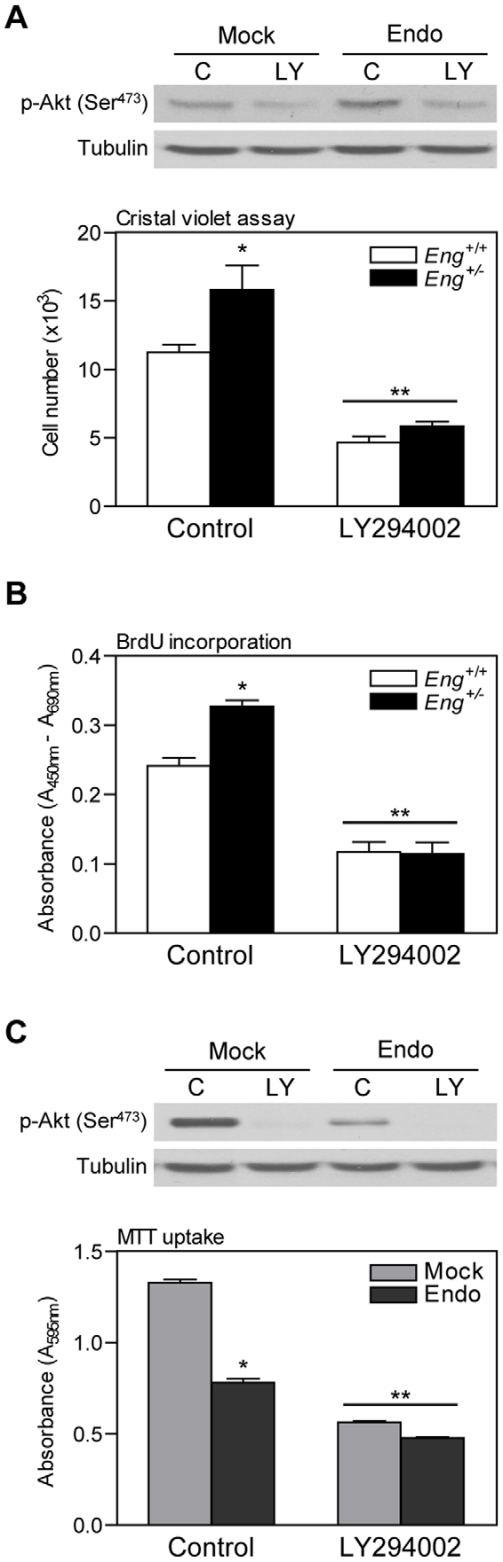
Effect of PI3K inhibition on endoglin-mediated different cell proliferation. (**A**) Cell number of MDF was analyzed by crystal violet assay after 10 μM LY294002 treatment for 4 days. Inhibition of Akt activation after LY294002 treatment was analyzed by western blot (**A**, upper panel). (**B**) BrdU incorporation of *Eng^+/+^* and *Eng^+/−^* MDF after LY294002 treatment was also analyzed. (**C**) Endo and Mock fibroblasts proliferation was assessed by MTT after 10 μM LY294002 treatment. Inhibition of Akt activation after LY294002 treatment was analyzed by western blot (**C**, upper panel). Mean+SEM is represented (n = 3). * p<0.05-significance of the difference between cells in control conditions, ** p<0.05-significance of the difference between LY294002 treatment and control conditions, Two-way ANOVA. Tubulin was used as loading control. A representative blot from three independent experiments is shown.

In order to determine the relationship between endoglin haploinsufficiency-induced proliferation and endoglin haploinsufficiency-induced Akt activation, we analyzed the effect of the PI3K inhibitor LY294002 on fibroblast proliferation. LY294002 has been shown to act as a highly selective inhibitor of PI3K, being an important tool for elucidating the biological role of the PI3K/Akt pathway [52], [53]. Whether the differences in cell proliferation found in endoglin-deficient cells will be a direct consequence of Akt activation, then, the treatment of these cells with the PI3K inhibitor, LY294002, would prevent the differences in endoglin-dependent cell proliferation. Consistent with the above results, in the absence of LY294002, the number of cells was higher in *Eng^+/−^* than in wild-type cells. Incubation with LY294002 resulted in an inhibition of both *Eng^+/+^* and *Eng^+/−^* cell proliferation and the number of cells was similar in both *Eng^+/+^* and *Eng^+/−^* cells (Figure 8A). Similar results were obtained after analysis of cell proliferation by BrdU incorporation (Figure 8B).

Furthermore, we studied the effect of LY294002 treatment on NIH3T3 cell proliferation in the presence or absence of endoglin overexpression. In the absence of LY294002, the number of cells, four days after plating, was lower in NIH3T3 cells overexpressing endoglin than in control cells. LY294002 treatment inhibited proliferation in both cell lines and the differences in proliferation disappeared (Figure 8C). Western blot analyses revealed that LY294002 at a dose of 10 μM was sufficient for the inhibition of Akt phosphorylation in both MDF and NIH3T3 cells (Figure 8A–C).

### Activated fibroblast accumulation in *Eng^+/−^* wounds is related to increased Akt phosphorylation

**Figure 9:**
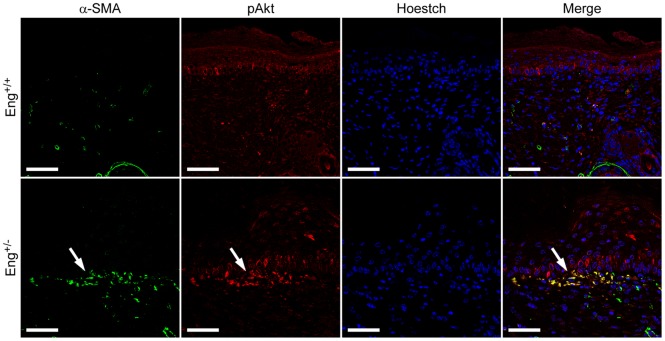
Histological analysis of Akt phosphorylation after wound healing. Immunofluorescence staining of alpha-smooth muscle actin (α-SMA) and phospho-Akt (pAkt) on Formalin-fixed Parafin-embedded wounds from *Eng^+/+^* and *Eng^+/−^* mice at day 12. Bars = 50 mm. Arrow = Colocalization of α-SMA and pAkt. A representative image from four independent experiments is shown.

In order to study in more detail the involvement of the PI3K/Akt pathway in the accumulation of activated fibroblasts *in vivo*, we analyzed Akt phosphorylation in 12-day-post-wounding *Eng^+/+^* and *Eng^+/−^* skin sections (Figure 9). Control skin sections did not display any particular distribution of pAkt, although the degree of Akt phosphorylation seemed to be higher in the basal epidermis than in the dermis. Interestingly, the distribution of Akt phosphorylation in the upper layer of the dermis of the *Eng^+/−^* scars was focused at the sites of α-SMA expression, coincident with the location of activated fibroblasts. Thus, α-SMA staining co-localized with Akt phosphorylation in *Eng^+/−^* but not in *Eng^+/+^* scars, indicating the direct relationship between endoglin haploinsufficiency, Akt phosphorylation and the maintenance of activated fibroblasts in mice skin.

## Discussion

The importance of endoglin in fibrosis has been widely accredited [11], [12], [13], [14], [15], [16], [17], [18], [19], [28], [29]. Fibrosis is the end result of a succession of events that occur after mechanical damage to the epithelium and/or endothelium [54]. Fibrotic diseases may be attributable to a variety of causes, but it is generally thought that an initiating injury event activates repair processes that aim to restore the original tissue architecture, and a failure to finely tune the repair process leads to persistent fibroblast activation and tissue destruction [55]. During physiological dermal wound healing, the activity of activated fibroblasts is terminated when the tissue is repaired. In pathological wound healing, however, activated fibroblasts persist and lead to tissue deformation and fibrosis [3], [4], [56]. The potential role of endoglin in fibroplasia during wound healing has been proposed [5] since a significant elevation in fibroblast-associated endoglin levels was observed between days 4 and 10 of wound healing [57]. To investigate the functional role of endoglin in wound healing induced fibrosis we have used mice heterozygous for endoglin (*Eng^+/−^*) and their wild-type littermates (*Eng^+/+^*) [31]. Endoglin knockout mice die at midgestation because of defective angiogenesis [7]. *Eng^+/−^* mice have allowed to analyze the involvement of endoglin in different processes such as angiogenesis [43], cardiovascular function [34] and tumor development [58], [59]. In this work, we show that *Eng^+/−^* mice exhibit persistence of activated fibroblasts in the wounds, as detected by α-SMA staining. In addition, primary cultured fibroblasts from these mice display higher expression of ECM-related molecules, increased proliferation and migration rates and diminished responses to apoptotic signals, whereas endoglin over-expression results in an inhibition of these processes. According to these results endoglin deficiency would lead to an increased fibrosis post-wounding. Although some studies suggest a profibrotic role for endoglin [13], [28], [29], our results are consistent with many previous works that demonstrate that endoglin acts as an antifibrotic molecule [14], [20], [21], [22], [23], [24], [25], [26], [27].

This antifibrotic effect of endoglin seems to be mediated by PI3K/Akt signaling pathway. Thus, under basal conditions Akt phosphorylation was increased in *Eng^+/−^* dermal fibroblasts and this effect was inhibited after endoglin overexpression. Akt/PKB is an intermediate signaling component of the PI3K pathway that is activated by phosphorylation in Thr and Ser residues and that is involved in several cellular processes, including growth, metabolism, reproduction, and life span [60], [61], [62]. In particular, Akt has been described as a fibroblast proliferation promoter [53], [63]. Here, we show that PI3K pathway inhibition reduced the faster proliferation of endoglin-haploinsufficient dermal fibroblasts to levels similar to those of control cells. Moreover, the differences in cell proliferation due to endoglin expression in NIH3T3 fibroblasts were abolished after PI3K inhibition. These results suggest that the difference in proliferation found in endoglin-haploinsufficient cells is a direct consequence of different Akt activation. Recently, the role of GSK-3β in wound healing has been analyzed [64]; GSK-3β is a downstream member of the PI3K pathway that is degraded after Akt-mediated phosphorylation. Interestingly, GSK-3β knock-down results in increased *in vivo* activated fibroblasts accumulation in the wound area and enhanced proliferation and migration *in vitro*. Our work, together with that of the above authors, highlights the relevance of PI3K/Akt pathway as an important mediator during wound healing and its importance in the regulation of activated fibroblasts accumulation after repair.

Whatever the mechanism of endoglin regulating Akt activation this effect seems to be independent of TGFβ1 signaling. It is widely accepted that endoglin has an important role regulating TGFβ signaling. Endoglin expression promotes TGFβ-mediated ALK1-Smad1/5 activation, in contrast with the classical ALK5-Smad2/3 activation. This model has mainly been described in endothelial cells [24], [45], and myoblasts [25], [26]. However, our study supports a previously proposed idea that endoglin has cellular effects independent of TGFβ [9], [49], [65], [66]. There are only a few works in the literature that relates endoglin and PI3K-Akt pathway and almost all of them refer a regulation of endoglin expression by PI3K-Akt pathway [67], [68], [69], [70], [71]. According with our results, very recently, *Lee et al.* shown the PI3K/Akt pathway as an endoglin target in regulating the stabilization of capillary sprouts and endothelial cell survival. They proposed a model in which GIPC could be the mediator coupling endoglin to the PI3K subunits and Akt at the plasma membrane and could be implicated in a differential response to TGFβ1 and BMP-9. In the presence of endoglin, TGFβ1 inhibits Akt phosphorylation with a modest enhancement by BMP-9 treatment [72]. Our data support these results as we also find a regulation of Akt phosphorylation depending on endoglin expression. Moreover, endoglin regulated PI3K/Akt signaling pathway could be a broad mechanism involved in the regulation of further physiologic processes and in different cell types.

In summary, our work analyzing the involvement of endoglin in activated fibroblasts accumulation may contribute to a better understanding of the pathophysiological processes of wound healing that give rise to fibrogenesis. Moreover, we proposed the PI3K/Akt pathway as the mechanism of action of endoglin on the regulation of post-wound healing fibrosis. Therefore, according to our results, endoglin and the PI3K/Akt pathway may be therapeutic targets for the treatment of fibrotic wounds.
